# A Combination of Green Tea, Rhodiola, Magnesium, and B Vitamins Increases Electroencephalogram Theta Activity During Attentional Task Performance Under Conditions of Induced Social Stress

**DOI:** 10.3389/fnut.2022.935001

**Published:** 2022-07-22

**Authors:** Neil Bernard Boyle, Louise Dye, Clare Louise Lawton, Jac Billington

**Affiliations:** School of Psychology, University of Leeds, Leeds, United Kingdom

**Keywords:** stress, green tea, rhodiola, magnesium, EEG

## Abstract

**Background:**

A combination of green tea, rhodiola and magnesium with B vitamins has previously been reported to significantly increase EEG resting state theta, attenuate subjective stress, anxiety and mood disturbance, and heighten subjective and autonomic arousal under acute psychosocial laboratory stress. Here we examine the capacity of green tea and rhodiola extract administered in combination or in isolation with magnesium and B vitamins to moderate spectral brain activity during attentional task performance under stress.

**Materials and Methods:**

One-hundred moderately stressed adults received oral supplementation of (i) Mg + B vitamins + green tea + rhodiola; (ii) Mg + B vitamins + rhodiola; (iii) Mg + B vitamins + green tea; or (iv) placebo, in a double-blind, randomised, placebo-controlled, parallel-group design (Clinicaltrials.gov: NCT03262376; 25/0817). Participants completed an attention switching and emotionally threatening attentional bias task after stress induction (Trier Social Stress Test). Spectral alpha and theta brain activity and event related potentials (ERPs) were recorded during cognitive task performance by electroencephalogram (EEG; BioSemi ActiveTwo 64 channel).

**Results:**

The combined treatment of Mg + B vitamins + green tea + rhodiola significantly increased frontal midline theta vs. placebo and rhodiola in isolation during the attention switching task, specifically in anticipation of a change in task performance parameter. The combined treatment also significantly increased contralateral theta activation in relation to viewing emotionally threatening images in the left (vs. placebo and rhodiola in isolation) and right parietal (vs. placebo) regions. Further, this treatment demonstrated significantly heightened ipsilateral left parietal theta activation in relation to viewing emotionally threatening images. The combined treatment attenuated a decrease in alpha power during the attentional bias task evident in comparator treatments, but this did not reach significance. No significant effects of treatments on behavioural performance or ERP were found.

**Conclusion:**

The combination of Mg + B vitamins + green tea + rhodiola increased spectral theta brain activity during the execution of two attentional tasks suggestive of a potential to increase attentional capacity under conditions of stress. Further examination of these ingredients in relation to attentional performance under stress is warranted to ascertain if functional benefits suggested by theta activation can be shown behaviourally.

## Introduction

Oscillatory brain activity measured by electroencephalogram (EEG) is an emerging marker for assessing the functional benefits of nutrient intake. Significant modulation of neural EEG activity has been demonstrated after acute intake of both green tea (1–3) and rhodiola (4). We have recently reported that combining green tea and rhodiola extract and administering with magnesium (Mg) and a B vitamin complex (B_6_, B_9_, and B_12_) significantly increased resting state frontal midline theta activity under stress conditions compared to placebo, and rhodiola extract combined with Mg and B vitamins (5); *post hoc* exploratory analysis also demonstrated the combined treatments superiority to green tea in isolation combined with Mg and B vitamins. Increased frontal midline theta is associated with a relaxed, alert state and anxiolytic action (6) suggesting the combination of these ingredients has the potential to enhance coping capacity and offer protection from the impairing effects of stress exposure. Further, the fully combined treatment was significantly more efficacious in the alleviation of the negative effects of stress exposure on subjective state. Here we report further data from this study on the combined effects of green tea, rhodiola extract, Mg and B vitamins on spectral frequency and event related potentials (ERPs) as measured by EEG during the completion of two attentional tasks under conditions of acute stress.

Attentional tasks sensitive to stress exposure and that engage neural processes previously shown to be moderated by green tea or rhodiola intake were selected for examination. Attention switching tasks typically require respondents to repeatedly perform a task on some trials then switch to another task when cued to do so, thus requiring the effortful suppression of a dominant or distracting response (1, 7). Attention switching performance is sensitive to impairment by stress (8). Further, acute intake of L-theanine – a constituent found almost exclusively in green tea – has been demonstrated to modulate alpha band activity associated with the deployment of attentional resources and suppression of distracting information (1, 9). Attentional bias tasks measure the degree to which attention is selectively focussed on a certain type of stimuli over another. Typically, threatening or rewarding stimuli are compared to neutral stimuli to determine the level of vigilance toward or avoidance of specific stimuli categories. Attentional bias performance is moderated by stress and anxiety (10). Rhodiola intake has also been shown to activate theta band activity whilst viewing emotional/threatening stimuli which has been interpreted as enhanced cognitive processing capacity (4). These demonstrated neural effects are indicative of potential functional benefits of green tea and rhodiola intake during the completion of attentional operations. Moreover, the previously demonstrated combined effects of these ingredients in the activation of EEG activity associated with a relaxed, attentive state and reduction of the subjective impact of stress exposure suggests a potential to offer direct protection from the impairing effects of stress by increasing stress coping capacity.

This randomised, placebo controlled trial examined the effects of a combination of green tea, rhodiola extract, Mg and B vitamins on attentional performance and associated spectral frequency EEG alpha and theta responses, and attention related ERPs, after acute stress provocation. The combined treatment was compared to placebo, and rhodiola or green tea combined with Mg and B vitamins in isolation to ascertain the potential capacity of the ingredients to affect behavioural attentional performance, and associated spectral brain activity, under stress. The combined treatment was expected to offer greater behavioural cognitive functional benefit which would be reflected in the associated EEG activity compared to placebo and treatments administering green tea and rhodiola in isolation.

## Materials and Methods

### Study Design

The study conformed to a double blind, randomised, placebo controlled, parallel group design comprising four treatment arms: (i) ***COMBINED:*** Mg + vitamins B_6_, B_9_, B_12_ + green tea + rhodiola extract; (ii) ***GREEN TEA*:** Mg + vitamins B_6_, B_9_, B_12_ + green tea; (iii) ***RHODIOLA:*** Mg + vitamins B_6_, B_9_, B_12_ + rhodiola extract; (iv) ***PLACEBO***. The design and primary and secondary outcomes of the study were pre-registered at Clinicaltrials.gov (NCT03262376; August 25th 2017). The study was approved by the University of Leeds, School of Psychology Research Ethics Committee (17-0235; granted October 30th 2017) and conducted according to the principles of Good Clinical Practice and the Declaration of Helsinki (11).

### Primary Outcomes

The effect of treatment on EEG spectral frequency oscillatory brain activity during the rested state [previously published (5)] and during completion of attentional tasks under conditions of stress exposure.

### Secondary Outcomes

The effect of treatment on subjective state, salivary cortisol, cardiovascular parameters [previously published (5)], cognitive performance and attentional event related potentials (ERPs).

### Participants

One hundred healthy adults (men = 36; women = 64) were recruited from the University of Leeds and local community. Eligible participants were identified from responses to an online eligibility screening questionnaire [*N* = 535; the full CONSORT diagram is previously published (5)]. Inclusion and exclusion criteria were as follows:

#### Inclusion Criteria

– Reporting moderate subjective stress levels [score of ≥13–≤25 on *stress* subscale of the Depression Anxiety and Stress Scale (DASS) (12)].
– ≥18–≤50 years of age (premenopausal if female indicated by self-reported lack of menopause symptoms or associated absence of period for 12 months+).
– BMI ≥18 and ≤30 kg/m^2^.
– Healthy [free from significant physical or psychiatric disorders (self-report)].

#### Exclusion Criteria

– Intake of prescribed medication except contraceptives.
– Hypertension (self-report or resting blood pressure >160/95 mmHg).
– Intake of regular nutritional supplementation.
– Smoking >5/day.
– Pregnant or lactating.
– Previous exposure to a laboratory stress protocol.
– Night-working/shift work.
– Recreational drug use.
– Previous brain surgery or severe brain injury requiring hospital treatment.
– Any form of neurological condition (e.g., epilepsy).

The eligibility criteria were confirmed at a face-to-face screening and written informed consent attained from all participants. An honorarium of £60 was paid for completion of the study protocol.

### Treatments

Magnesium and B vitamins were administered in a single pressed tablet form [Mg (150 mg elemental) + vitamins B_6_ (0.7 mg), B_9_ (0.1 mg), B_12_ (0.00125 mg)]. Teadiola^®^ (Sanofi) – a commercially available combined green tea/rhodiola extract formulation – was employed for the green tea and rhodiola treatments. To permit administration of the two ingredients, both combined and in isolation, the manufacturer was requested to split the formulation into separate treatments: dry extracts of green tea leaf [*Camellia sinensis* L. 125 mg containing 40% L-theanine (50 mg)], and rhodiola root (*Rhodiola rosea* L. 222 mg, or 1,887 mg equivalent plant) standardised in rosavins and salidrosides. To ensure blinding these separate treatments were administered in opaque capsule form. Identical placebo equivalent forms (cellulose crystalline) were produced for all treatments. All treatments were manufactured by an affiliate of Sanofi.

### Randomisation

Respondents were allocated a 6 digit/string ID code^1^ at the face-to-face screening visit. Eligible respondents were subsequently randomly allocated to treatment by an independent statistician using an adaptive randomisation scheme to ensure a balanced representation of age and gender across the treatment arms (13).

### Blinding

Participants were allocated to treatment arms by the independent statistician using 4 treatment codes generated by an independent technician at Sanofi. The treatments were prepared at the Leeds site by a laboratory manager not directly involved in testing. All treatments were supplied in identical forms and identifiable to the experimenters only by a packing number.

Final data sets were locked upon completion of data collection and initial EEG processing and duplicate copies logged. The Leeds team and statistician remained blinded until data locking and completion of primary statistical analyses. Treatments codes were broken to complete *a priori post hoc* comparisons as per the fixed hierarchical statistical analysis approach employed (detailed in section “Statistical Analyses”).

### Measures

#### Cognitive Tasks

Two attentional cognitive tasks were employed to permit EEG measurement of neural processing and cognitive performance under stress, and to assess the effects of treatment on these outcomes. Both cognitive tasked were presented using PsychToolBox plugin for MATLAB on a desktop computer and an iiyama Prolite B2483HS 24″ display (1920 × 1080 full HD 1080p, 2.1 megapixel, 60 Hz refresh rate).

##### Attention Switching Task

**FIGURE 1:**
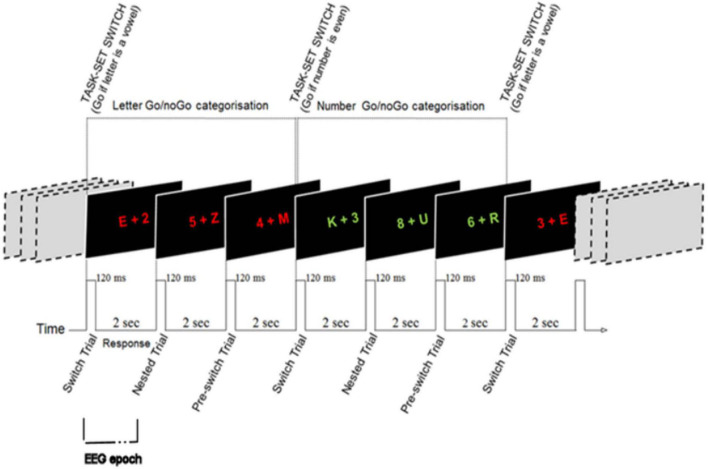
Attention switching task stimulus configuration [adapted with permission from Wylie et al. (7)] showing seven consecutive trials/EEG epochs.

The attention switching task was originally develop by Wylie et al. (7) and combines an attention switching paradigm with a Go/No-Go task. Letter-number pairs were presented on a horizontal plane in the centre of the screen for 2 s (120 ms inter-stimulus delay). Each character was 1° to the left or right of the central fixation point (randomly determined). The letters were taken from a set of 4 vowels (A, E, I, and U) and four consonants (G, K, M, and R). The numbers were taken from a set of 4 even (2, 4, 6, and 8) and 4 odd (3, 5, 7, and 9) numbers. Letter-number pairs were presented in one of two alternating colours every three trials. Respondents were required to make a Go/No-Go choice based upon the colour of the letter-number pair with the change in colour cueing the switch in task-set. For example, when the letter-number pairs were red, respondents were required to respond when the letter was a consonant (Go), but not when the letter was a vowel (No-Go). Alternatively, when the letter-number pairs switched to white, respondents were required to respond when the number was odd (Go), but not when the number was even (No-Go). *Switch* trials were the first letter-number pairs presented after the task-switch (i.e., the Go/No-Go colour switch). *Nested* and *pre-switch* were the subsequent repeat trials within the same task-set. A total of 216 trials were presented (72 three trial task sets). Performance was measured by reaction time (RT) and accuracy across each trial type. See Figure 1 for stimulus configuration.

##### Attentional Bias Task

**FIGURE 2:**
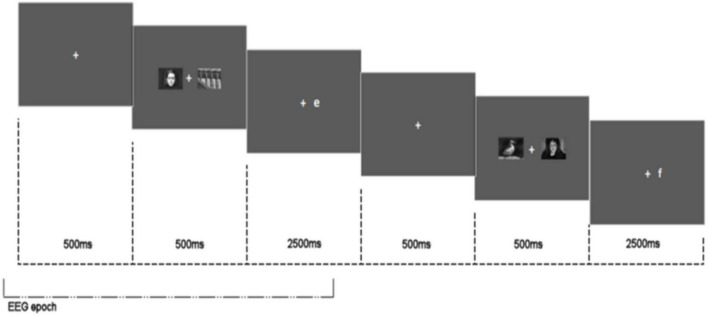
Attentional bias task stimulus configuration showing two consecutive trials and EEG epoch.

A simple dot probe task comprising neutral (*n* = 24) and emotionally threatening (*n* = 24) images taken from the International Affective Picture System (IAPS) (14) was employed. A central fixation cross was shown for 500 ms followed by two images – one neutral and one threatening – presented simultaneously on a horizontal plane in the centre of the screen 1.5° to the left or right of the central fixation point. The images measured 150 mm width by 80 mm height and were shown for 500 ms. After a 500 ms cue-target delay, a target – the letter E or F – appeared on the left or right in the centre of the location previously occupied by one of the displayed images. Participants were asked to press E or F on the keyboard dependent upon which letter target was displayed. The presentation of the target letter coincided with the position of the emotionally threatening image (congruent trial), or the position of the neutral image (incongruent trial) with a 50% probability. The pairing of neutral and emotionally threatening images was held constant for all participants. Each stimulus image pair was shown three times across the task. A total of 144 trials were presented comprising 72 congruent and 72 incongruent trials. Target type (E or F), location of emotionally threatening/neutral image and target (left or right of fixation) were all counterbalanced and trials were presented in random order. Participants were instructed to look at the fixation cross and discriminate the target as quickly and accurately as possible. A congruency score was calculated by subtracting mean RT for congruent trials from incongruent trials. A congruency score below zero is indicative of attentional avoidance and scores above zero of attentional vigilance. See Figure 2 for stimulus configuration.

### Electroencephalogram Methodology

Electroencephalogram was recorded continuously with a BioSemi ActiveTwo EEG system (BioSemi, Amsterdam) from 64 electrodes at a 1,024 Hz sampling rate. Four electrooculograms (EOG) electrodes were used to record eye movements and were placed lateral and dorsal to each eye. Two EOGs were placed on the mastoids. EEG recordings comprised four separate blocks: two resting state measures (580 s), attention switching task (550 s), and attentional bias task (530 s). All tasks were completed in sequential order in one session. The resting state data is reported elsewhere (5).

Prior to analysis, EEG data were pre-processed in MATLAB using both EEGLAB (15) and Fieldtrip (16). Data were initially down sampled to 512 Hz. All participants’ data were checked by eye and exceptionally bad channels removed. Continuous data was then re-referenced to the average of all 64 channels (with exception of removed bad channels) and data destined for spectral analysis were high-pass finite impulse response (FIR) filtered using a 1 Hz cut-off. Independent component analysis (ICA) (17) was used to identify eye-movement components in the data. Prior to spectral analysis, data was high-pass finite impulse response (FIR) filtered using a 0.1 Hz cut-off, and ICA components derived from the 1 Hz filtering set were used to remove eye-movement artefacts from 0.1 Hz filtered set. Data were segmented into epochs and baseline corrected using the following methodology:

#### Attention Switching Task

Switch, nested and pre-switch trials were considered individual epochs. Epochs were extracted −0.1 to 1.7 s to trial onset (appearance of stimulus). Baseline period: −100 to −20 ms (epoch shown in Figure 1).

#### Attentional Bias Task

Each trial (comprised of crosshair, picture presentation, and response letter presentation) was considered an individual epoch. Epoch length −0.1 to 3.3 s relative to crosshair onset. Baseline period: −100 to −20 ms (epoch shown in Figure 2).

After defining epochs a MATLAB implementation of the Fully Automated Statistical Thresholding for EEG Artefact Rejection [FASTER (18)] was used to remove artefacts in the data. This included (1) thresholded interpolation of bad channels via identification of global and local artefacts in channel data and (2) removal of bad trials on the basis of remaining eye and muscle movement noise and voltage spikes. Spectral power was estimated across each epoch in the range of 2–40 Hz in 0.1 Hz steps using a Hann taper with a variable time window (four cycles at each frequency). Frequencies of specific interest – alpha (8–12 Hz) and theta (4–7 Hz) – were extracted for statistical analysis. Alpha and theta EEG frequency data were extracted for each cognitive task using the following methodology:

##### Attention Switching Task

Alpha and theta values were extracted from each trial epoch – switch, nested and pre-switch trials – and averaged for each participant.

##### Attentional Bias Task

Alpha and theta values were extracted from each epoch and further binned into 250 ms time bands to permit comparisons of alpha/theta activity over the course of the trial.

### Event-Related Potential Methodology

High-density event-related potentials (ERPs) were extracted from frontal and parietal sites during completion of the attention switching task. The componentry of the ERPs was extracted using the following electrode configuration: Left Frontal: “AF3,” “F3,” “F1,” Right Frontal: “AF4,” “F2,” “F4”; Left Parietal: “PO3,” “PO7,” “O1,” Right Parietal: “PO4,” “PO8,” “O2.” To permit ERP componentry identification, data from these site-specific electrodes were collapsed across treatment arms and averaged into a single average waveform for the left and right hemisphere sites for each trial type (switch, nested, and pre-switch). The waveforms were visually inspected and their componentry broadly defined in accordance with the components originally identified for this specific attention switching task by Wylie et al. (7) The ERP components extracted for statistical analyses are shown in Table 1.

**TABLE 1:** Attention switching task event related potential component summary [with onset and offset timings (ms)] by region.

Region	Component	P1	N1	P2	LP1	LP2			
Frontal	Onset (ms)	0.1284	0.2340	0.2827	0.8	1.4			
	Offset (ms)	0.1684	0.2740	0.3227	1.3	1.650			
	**Component**	**P1**	**N1**	**P2**	**N275**	**P400**	**P550**	**LP1**	**LP2**
Parietal	Onset (ms)	0.058	0.116	0.218	0.274	0.370	0.544	0.8	1.4
	Offset (ms)	0.098	0.156	0.258	0.314	0.570	0.564	1.3	1.65

### Stress Protocol

The Trier Social Stress Test (TSST) was employed to experimentally induce acute stress. The TSST protocol has been defined elsewhere (19). Briefly, participants completed two extemporaneous 5 min socially evaluated tasks – presenting themselves as a job candidate and completing a maths subtraction task – in front of an unresponsive panel of two female confederates.

### Procedure

**FIGURE 3:**
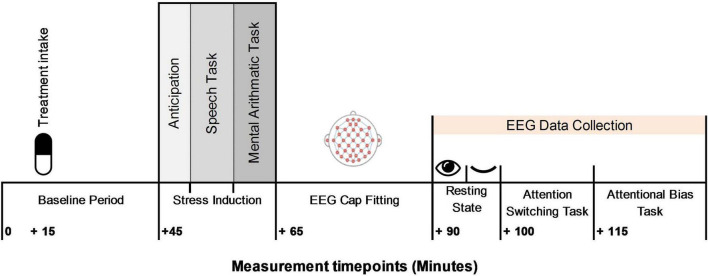
Procedural timeline.

The study was undertaken at the Human Appetite Research Unit (HARU), School of Psychology, University of Leeds, United Kingdom. All participants completed the study protocol between 11 and 3 pm, fasted 1 h prior to commencement of the study protocol, and avoided alcohol and vigorous exercise 24 h prior to the visit. The test day procedural timeline is shown in Figure 3. Upon arrival participants relaxed in the testing cubicle. A number of physiological and subjective measures were collected throughout the visit. These measures are reported elsewhere (5). After 15 min the allocated treatment was administered with water and consumption witnessed and checked by the experimenter. Participants relaxed in the cubicle for 30 min before being taken to a separate room and introduced to the TSST task. Participants returned to the testing cubicle for a 5 min anticipation period. Participants then completed the TSST (lasting ∼ 10 min). Following stress induction participants were taken immediately to the EEG laboratory for fitting of the BioSemi and a clear signal established, participants completed 10 min of resting state measures. The attentional switching and attentional bias task were then undertaken in sequential order.

### Statistical Analyses

All statistical analyses were performed using SAS (Statistical Analysis System, Version 9.2; SAS Institute, Inc., Cary, NC, United States) and Fieldtrip (16). Cognitive performance outcome residual plots were inspected for deviations from normality and residual outlying data points removed (±2.58 SD). One participant was removed from the study and replaced due to a fault in the EEG system preventing measurement. The primary analyses were performed in the randomised population, defined as all randomised subjects who received at least one dose of the treatment and for whom a reliable EEG signal could be obtained.

A sample size power calculation was conducted based upon previous evidence of the modulation of resting state and attentional performance-related EEG alpha wave activity by L-theanine (1–3, 9). Reported effect sizes ranged from 0.803 to 1.141 (Cohen’s *d*). Assuming an effect size of 0.9, α = 0.05, and a power of 80%, it was estimated that a sample of 25 participants per arm (100 total) would be required to find an effect of this magnitude.

For all EEG analysis (spectral frequency and ERP), individual trials for each condition were averaged for each participant prior to statistical analysis in Fieldtrip or mean extraction for ROI analysis. A whole brain approach was initially adopted comparing oscillatory activity between the COMBINED vs. PLACEBO treatments as a primary analysis step. For this, independent sample *T*-tests (Monte Carlo approach 500 random permutations for all electrodes) were employed in Fieldtrip (16) to compare oscillatory alpha and theta power magnitude (μV) across the brain. Secondary regions of interest (ROIs) analyses were carried out on ROIs in which a significant difference was observed in this primary analysis step (*p* < 0.05). ROIs selected for further analyses were informed by the whole brain result but with ROIs defined *a priori* on the basis of previous literature. Regions were defined in this way in order to prevent circularity and bias in data analysis (20) and analysed using a six-sequence mixed effect ANCOVA in SAS as per the procedure outlined below.

A mixed-effects ANCOVA was employed with treatment and gender entered as fixed model effects and participant ID entered as a random effect. Age, gender, BMI, trait anxiety (STAI-Y2), and DASS stress score were entered as covariates in all models and removed or retained based on the Akaike Information Criterion (AIC). To control the family-wise type 1 error rate, a fixed sequence testing procedure (six-steps) was used to assess the effect of each treatment arm within each primary outcome. The six-step sequence adhered to the following order: (1) COMBINED vs. PLACEBO; (2) COMBINED vs. RHODIOLA; (3) GREEN TEA vs. PLACEBO; (4) RHODIOLA vs. PLACEBO; (5) COMBINED vs. GREEN TEA; and (6) RHODIOLA vs. GREEN TEA. The significance at a level of 0.05 was tested only if the results of the previous step reached significance. Secondary endpoints related to the secondary objectives were also tested using the same fixed sequence procedure. Exploratory (*post hoc*) analyses of the comparisons not reached in the fixed sequence due to non-significance of previous steps are briefly summarised in the discussion for exploratory purposes only and to aid future investigation of the ingredients.

## Results

A descriptive summary of the final sample is shown in Table 2. One way ANOVAs revealed no significant differences between the study conditions across the participant characteristics of Age [*F*(3,96) = 2.318, *p* = 0.08], BMI [*F*(3,96) = 0.359, *p* = 0.78], STAI-Y2 [(trait anxiety]; *F*(3,96) = 2.185, *p* = 0.10], and DASS Stress score [*F*(3,96) = 0.906, *p* = 0.44].

**TABLE 2:** Summary characteristics of sample receiving each treatment.

Treatment	*N*	Sex	Age	BMI	STAI-Y2	DASS stress score
			
			*Mean (SEM)*			
Green tea	25	16F 9M	26.44 (1.57)	22.9 (0.56)	38.72 (1.46)	16.6 (0.68)
Rhodiola	25	17F 8M	26.2 (1.13)	23.25 (0.63	40.48 (1.24)	15.68 (0.63)
Combined	25	14F 11M	22.2 (0.74)	22.93 (0.52)	42.48 (1.43)	17.12 (0.74)
Placebo	25	17F 8M	25.56 (1.55)	23.69 (0.56)	43.12 (1.37)	15.96 (0.66)

### Electroencephalogram Outcomes

#### Attention Switching Task

##### Alpha

Whole brain analysis of oscillatory alpha band activity revealed no significant differences across the electrode sites between the COMBINED and PLACEBO treatments during execution of the attention switching task. Heightened alpha at FCz and surrounding electrodes was evident in the COMBINED vs. PLACEBO treatment but this comparison did not reach significance (*p* > 0.05). Accordingly, no ROI analyses were undertaken.

##### Theta

**FIGURE 4:**
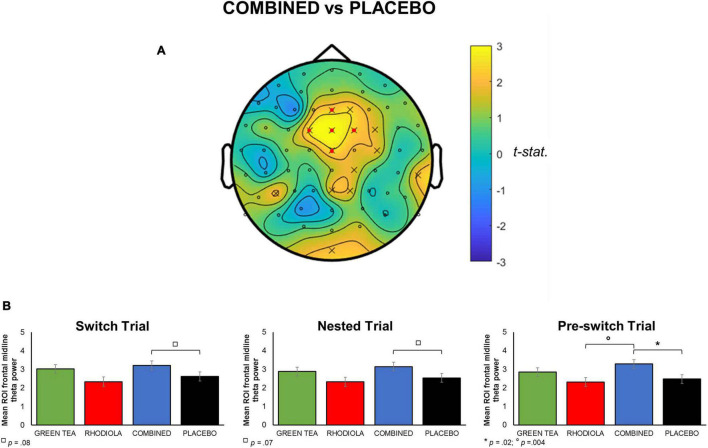
**(A)** Map of t-statistic comparison of oscillatory theta band activity during attention switching task in COMBINED vs. PLACEBO treatments (positive values indicate COMBINED > PLACEBO in comparison). *A priori* defined ROI midline frontal electrodes shown in map by 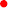. **(B)** Mean averaged ROI (SEM) frontal midline theta for each attention switching trial by treatment.

Whole brain analysis of oscillatory theta band activity revealed significantly heightened theta activity during the execution of the attention switching task (*p* < 0.05). This effect predominantly consisted of an overall heightened frontal midline theta in the COMBINED vs. PLACEBO treatment during performance of the attention switching task (Figure 4A). To permit further analyses, frontal midline theta values were extracted from channels “Cz,” “Fz,” “FCz,” “FC1,” “FC2,” and combined and averaged separately for switch, nested and pre-switch trials in each treatment. The COMBINED treatment heightened frontal midline theta compared to the PLACEBO during execution of the three attention switching trials (Figure 4B). This increased theta in the COMBINED treatment reached significance during the pre-switch trials, *t*(95) = 2.46; *p* = 0.02. The COMBINED treatment also significantly heightened frontal midline theta during pre-switch trials compared to RHODIOLA treatment, *t*(95) = 2.93; *p* = 0.004. No significant difference between the GREEN TEA treatment and PLACEBO was found for pre-switch trials. Accordingly, no further comparisons are reported.

#### Attentional Bias Task

##### Alpha

**FIGURE 5:**
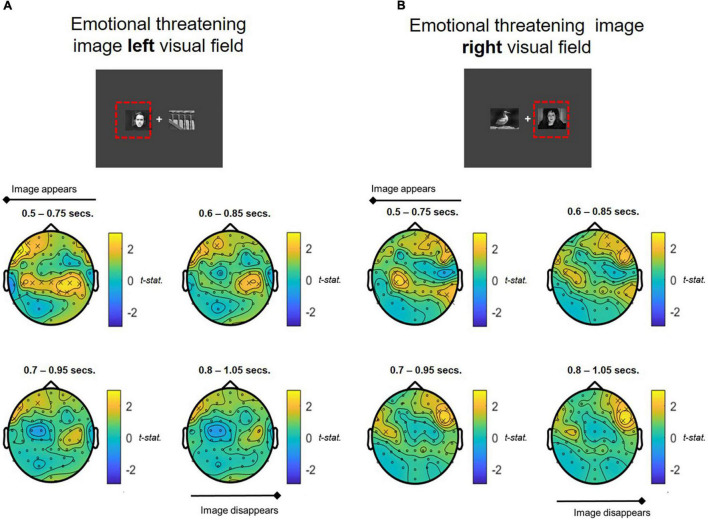
T-statistic map of alpha band power differences between COMBINED and PLACEBO treatments during presentation of emotionally threatening images shown in the **(A)** LEFT and **(B)** RIGHT visual fields. Values > 0 indicate COMBINED treatment alpha values are higher than PLACEBO treatment. Significant electrodes (*p* < 0.05) marked with an *x*. Each figure depicts a series of *T*-tests along the time course of the presentation of the images (0.5–1.05 s, in 0.1 s steps, 0.25 s sample widths).

Whole brain analyses of oscillatory alpha band activity over the time course of the attentional bias task revealed significant differences in alpha power in response to stimulus presentation compared to baseline in the COMBINED treatment vs. PLACEBO. There was a strong contralateral central-parietal alpha increase when emotionally threatening images were presented in either the left and right visual fields in the COMBINED treatment (Figures 5A,B). The COMBINED treatment also demonstrated heightened ipsilateral alpha activity in the central-parietal cortex for left presented emotionally threatening images (Figure 5A), but not right. For both left and right presented emotionally threatening images, there was significantly greater ipsilateral alpha activation in the frontal cortex in the COMBINED treatment compared to PLACEBO.

**FIGURE 6:**
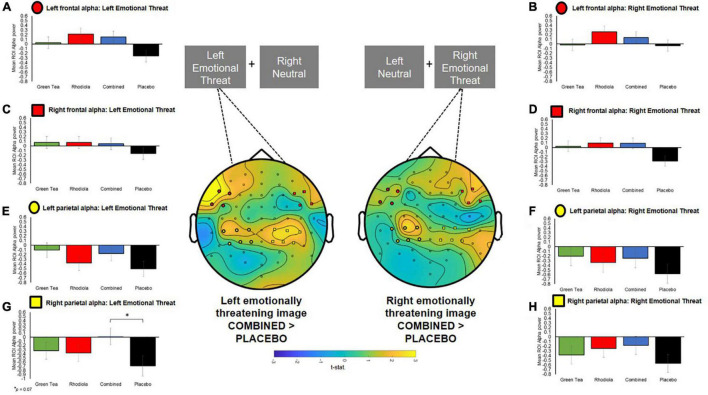
Central figure: T-statistics maps of alpha difference between COMBINED and PLACEBO treatments 0.5–0.75 s after presentation of the images, distinguished by left and right emotionally threatening image presentation. Positive values in maps indicate COMBINED > PLACEBO. ROI electrodes symbols shown on maps and in respective summary bar charts. **(A–D)** Ipsilateral and contralateral frontal *a priori* defined ROI alpha band power when emotionally threatening image shown on left and right. **(E–H)** Ipsilateral and contralateral centro-parietal *a priori* defined ROI alpha band power when emotionally threatening image shown on left and right.

To permit further exploration of the data, contralateral frontal and centro-parietal ROIs corresponding with the locus of alpha activity were extracted using the following configurations, representative of both frontal and parietal brain regions: Right Frontal: channels “F6,” “F8,” “FT8,” “FC6”; Left Frontal: channels “F5,” “F7,” “FT7,” “FC5”; Right Parietal: channels “CP2,” “CP4,” “CP6,” “P2,” “P4,” “P6”; Left Parietal: channels “CP1,” “CP3,” “CP5,” “P1,” “P3,” “P5” (ROIs are shown mapped on the scalp in Figure 6). ROI analyses were undertaken on spectral activity during the temporal response peak between 0.5 and 0.75 s immediately after the presentation of the images.

The COMBINED treatment increased contralateral right centro-parietal alpha power when viewing emotionally threatening images presented in the left visual field but this did not reach significance (*p* = 0.07; Figure 6). Since no primary comparisons were significant no further comparisons are reported.

##### Theta

**FIGURE 7:**
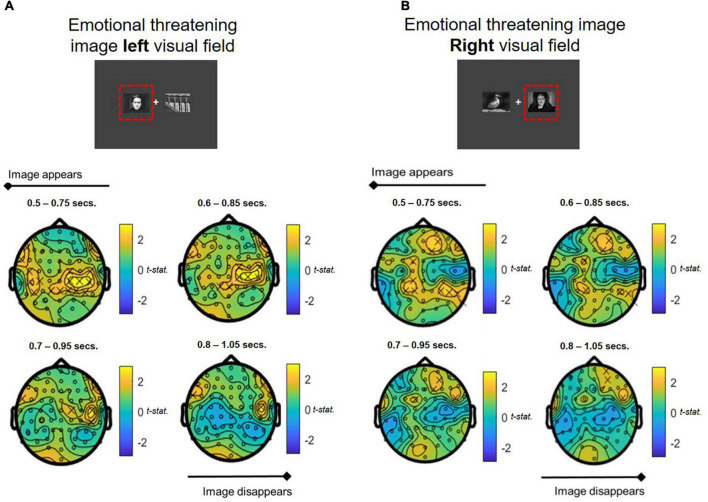
Theta band power differences between COMBINED and PLACEBO treatments during presentation of emotionally threatening and neutral images when stress images shown in **(A)** LEFT and **(B)** RIGHT visual fields. Values > 0 indicate COMBINED treatment theta values are higher than PLACEBO treatment. Significant electrodes (*p* < 0.05) marked with an *x*. Each figure depicts a series of *T*-tests along the time course of the presentation of the images (0.5–1.05 s, in 0.1 s steps, 0.25 s sample widths).

Whole brain analyses of oscillatory theta band activity over the time course of the attentional bias task revealed significant differences in theta power in response to stimulus presentation compared to baseline in the COMBINED treatment vs. PLACEBO. Significantly heightened theta activation was shown in ipsilateral and contralateral central-parietal regions in the COMBINED treatment during presentation of emotionally threatening images in the left and right visual fields (Figures 7A,B). The effect was more pronounced in contralateral regions. This central-parietal effect in the COMBINED compared to PLACEBO treatment declined over the course of the image presentation, becoming more focussed in right frontal regions, particularly when emotionally threatening images were presented in the right visual field (ipsilateral activation; Figure 7B).

**FIGURE 8:**
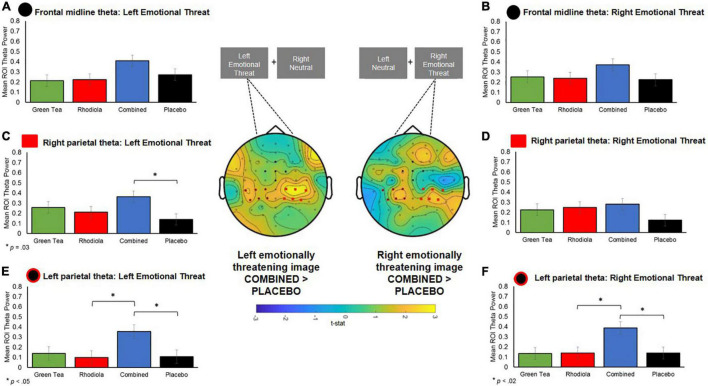
Central figure: T-statistic maps of spectral theta activity difference between COMBINED and PLACEBO treatments 0.5–0.75 s after presentation of the images, distinguished by left and right emotionally threatening image presentation. Positive values in maps indicate COMBINED > PLACEBO. ROI electrodes symbols shown on maps and in respective summary bar chart. **(A,B)** Theta band activity in *a priori* defined ROI midline frontal region for left and right presented images. **(C,D)** RIGHT parietal *a priori* defined ROI theta activity when emotionally threatening image shown on left and right. **(E,F)** LEFT parietal *a priori* defined ROI theta activity when emotionally threatening image shown on left and right.

To permit further analyses, frontal midline and left and right parietal ROIs corresponding with the locus of theta activity were extracted using the following configurations: Frontal midline: channels “Cz,” “Fz,” “FCz,” “FC1,” “FC2”; Left Parietal: channels “C1,” “C3,” “C5,” “CP1,” “CP3,” “CP5”; Right Parietal: channels “C2,” “C4,” “C6,” “CP2,” “CP4,” “CP6” regions (ROIs are shown mapped on the scalp in Figure 8). These analyses were undertaken on spectral activity during the temporal response peak between 0.5 and 0.75 s immediately after the presentation of the images.

No significant differences in theta activation were shown between COMBINED and PLACEBO treatments in the frontal midline region (*p* > 0.05; Figures 8A,B). Accordingly, no further comparisons are reported for this region.

Significantly greater contralateral theta activation was shown in the left parietal region in the COMBINED treatment when emotionally threatening images were shown in the right visual field compared to PLACEBO, *t*(95) = 2.94, *p* = 0.02, and RHODIOLA, *t*(95) = 2.91, *p* = 0.02 (Figure 8F), treatments. The COMBINED treatment also demonstrated significantly greater contralateral theta activation in the right parietal region when emotionally threatening images were shown in the left visual field compared to PLACEBO, *t*(96) = 2.77, *p* = 0.03 (Figure 8C). Additionally, the COMBINED treatment demonstrated significantly greater ipsilateral theta activation in the left parietal region when emotionally threatening images were shown in the left visual field compared to PLACEBO, *t*(96) = 2.64, *p* = 0.05, and RHODIOLA, *t*(96) = 2.74, *p* = 0.04, treatments (Figure 8E).

### Cognitive Performance

#### Attention Switching Task

**FIGURE 9:**
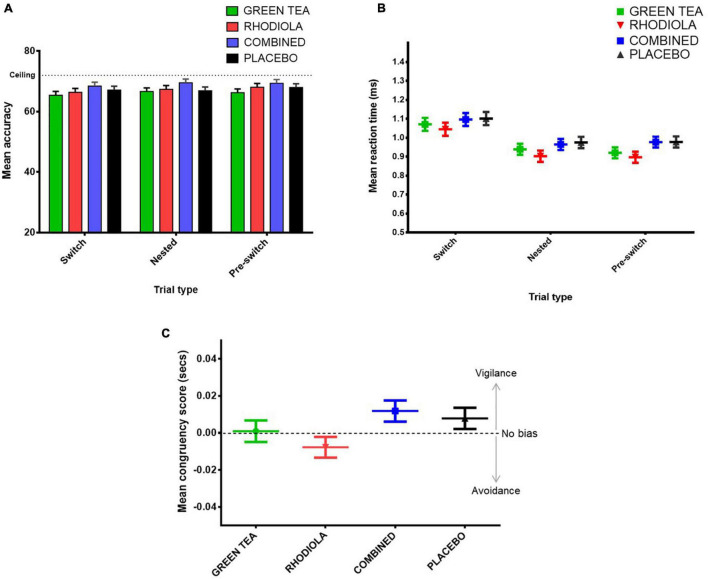
Attention switching task **(A)** mean accuracy and **(B)** RT (ms) by trial type and treatment, and **(C)** attentional bias task attentional congruency score (incongruent trials RT – congruent trials RT) by treatment.

The expected pattern of lower accuracy and slower RT on switch compared to repeated trials (nested and pre-switch) was demonstrated across the sample as a whole. However, no significant differences in accuracy or RT performance between trials were found between the COMBINED and PLACEBO treatments (Figures 9A,B). Accordingly, no further comparisons are reported.

#### Attentional Bias Task

No significant difference between congruent RT, incongruent RT or congruency score was shown between the COMBINED and PLACEBO treatments. Accordingly, no further comparisons are reported. Inspection of the congruency score data (Figure 9C) suggests the COMBINED and PLACEBO treatments show a pattern of attentional vigilance (score > 0), RHODIOLA treatment attentional avoidance (score < 0), and GREEN TEA treatment no bias (score = ∼0). However, all congruency scores fall within a range about zero of 25 ms. The validity of congruency scores below 25 ms has been questioned and a congruency score between −25 and +25 ms suggested as an appropriately conservative criterion to indicate a lack of attentional bias (21).

### Event Related Potentials

No significant differences between the COMBINED and PLACEBO treatments were shown across any of the extracted right or left frontal or parietal ERP components (*p* > 0.05). Accordingly, no further analyses are reported. Inspection of the overall ERP waveform collapsed across all treatments showed that the typical attentional ERP components expected to be shown during the completion of an attention task were demonstrated (e.g., P1, N1, and P2). However, the well-characterised ERP differentiation (7) between switch, nested and pre-switch trials typically observed in this attention switching paradigm was not demonstrated. Differentiation in ERPs for each trial type during this task typically become evident in frontal regions from ∼450 to 470 ms and parietal regions from ∼ 230 ms and show further sustained differentiation in the slow late potentials (LP1 and LP2) (7); this was not replicated here.

## Discussion

This study examined the capacity of green tea and rhodiola extract when administered in combination or in isolation with magnesium and B vitamins to moderate spectral brain activity during attentional task performance after acute stress provocation. As previously reported for frontal midline theta activity and subjective responses to acute stress provocation (5), the combination of these ingredients was shown to induce significantly greater effects, particularly in relation to theta activity. The study findings are discussed in relation to each attentional task employed below.

### Attention Switching

Overall, spectral EEG analysis during the attention switching task showed a trend for frontal midline theta to increase in the COMBINED treatment compared to the PLACEBO treatment, with the RHODIOLA and GREEN TEA treatments showing intermediate effects. This trend of heightened theta in the COMBINED treatment reached significance during pre-switch trials vs. PLACEBO and RHODIOLA treatments. Thus, demonstrating a trial specific heightened frontal theta activation in the COMBINED treatment during execution of the attention switching pre-switch trials. Contrary to this, alpha band spectral activity showed no treatment difference during the attention switching task.

Frontal theta activity has been implicated in the operation of numerous domains of cognitive function (6). Heightened frontal theta activity is most commonly associated with executive function and working memory performance, but also correlates to a diverse range of cognitive processes including: error processing (22), attention (23), and episodic memory (24). Tasks typically shown to increase frontal midline theta are those characterised by non-specific, focussed or sustained attention or concentration, or resistance to intrusion (6). This association with a broad range of higher-order cognitive functions has led to the proposal that frontal theta is the putative “language” of a prefrontal network, in which action monitoring and top-down control mechanisms interact to optimise performance (25, 26). Heightened frontal midline theta may be particularly relevant to the realisation of a need to exert greater cognitive control, for example, when required to implement adaptive control in situations incorporating conflicting stimulus-response requirements (27). This may account for the observed increased heightened frontal midline theta in the COMBINED treatment reaching significance in the pre-switch trials, due to the anticipated need to exert greater cognitive control for the impending switch in No-Go response on the following switch trial. This heightened frontal midline theta in the COMBINED treatment is indicative of enhanced processing capacity during the execution of the attention switching task. Since performance on attention switching tasks is vulnerable to the impairing effects of stress (8), this effect may be due to the previously demonstrated capacity of the COMBINED treatment to induce a relaxed focussed state and reduce subjective rating of stress and anxiety (5).

Evidence of potential theta activity-related enhanced attentional capacity in the COMBINED treatment was not reflected in behavioural performance on the attention switching task. The COMBINED treatment demonstrated a tendency toward higher accuracy and slower RTs – a cognitive performance pattern previously associated with heightened frontal midline theta (28) – but no significant performance differences between treatments were found. The attention switching task may not have been sensitive enough to demonstrate any potential enhancing effects of increased attentional capacity suggested by increased theta activation. However, this task employs cognitive processes closely associated with frontal theta activity – namely: focussed attention, executive control, resistance to intrusion – and is therefore a prime candidate task to confirm any positive behavioural effects of treatment. Attention switching tasks have also been previously shown to be sensitive to the enhancing effects of micronutrient interventions [e.g., green tea (29)]. However, the employment of alternative tasks may better demonstrate the potential behavioural enhancements of the COMBINED treatment.

The COMBINED treatment did not significantly moderate alpha activity during the attention switching task despite evidence of constituent ingredients previously being shown to increase alpha activity during attentional performance [L-theanine (1–3)]. Alpha activity has traditionally been associated general alertness and arousal states (30). A role for alpha has further been proposed in operation of selective attentional mechanisms (31); specifically, during attentional processing that requires the inhibition of non-essential/distracting stimuli (1). However, existing evidence of alpha activity involvement in selective attentional processing, and the capacity of green tea constituents to moderate this activity, primarily relates to the anticipatory suppression of distracting visual stimulation during the switching of attention between sensory modalities, particularly between visual and aural stimuli (1, 32). These findings were specific to the inferior and posterior parietal cortex, suggesting a principal engagement with visual processing regions (32). Therefore, the attention switching task employed here may not have modulated alpha activity due to less engagement of visuo-spatial attentional processes. Further, the capacity of green tea constituents to moderate alpha activity may be specific to the attentional switching tasks that primarily engage visuo-spatial processing.

### Attentional Bias

Overall, whole brain spectral EEG analysis during the attentional bias task showed a trend for theta to be higher for the COMBINED compared to PLACEBO treatment. This effect was predominantly seen in the contralateral (and sometimes ipsilateral) central parietal area. ROI analysis confirmed that this was a strong effect, with left and right images eliciting significant contralateral activation and ipsilateral in the right parietal region a short time after the emotionally threatening images were presented. The COMBINED treatment elicited significantly greater contralateral left parietal theta activation vs. PLACEBO and the RHODIOLA treatments and contralateral right parietal theta activation vs. PLACEBO treatment. *Post hoc* analyses showed the COMBINED treatments also significantly heightened left parietal contralateral theta activation vs. the GREEN TEA treatment (*p* = 0.02); suggesting the combination of the ingredients may be crucial in the observed effects. Significantly higher ipsilateral left parietal activation was also shown in the COMBINED treatment vs. PLACEBO and RHODIOLA treatments.

The attentional processing of affective stimuli is prioritised by the visual attention system to permit prioritised perceptual processing of threat-relevant stimuli – whilst paying less attention to non-threat relevant stimuli (33). This permits the rapid preparation of adaptive behavioural responses, such as avoidance of threat. Increased theta activation is associated with the viewing of affective stimuli such as emotional or threatening images, and facial expressions (34). Early theta activity in response to viewing threatening stimuli is commonly shown in the occipital and parietal-occipital lobes with later responses focussed in the frontal region (35–37); a regional and temporal specificity replicated here. These findings suggest the COMBINED treatment enhanced attentional processing function during an emotionally threatening attentional task. The greater contralateral activation – relative to the presentation of emotionally threatening images in the left and right visual field – suggest this effect was most pronounced for emotionally threatening stimuli. Such attentional vigilance to threatening stimuli is known to be heightened by stress states, with stress induction shown to narrow attentional processes toward threat salient stimuli (38, 39). However, we have previously reported the COMBINED treatment significantly attenuated subjective stress immediately prior to the attentional bias task (5), so a heightened stress response does not account for the difference in theta activation observed in the COMBINED treatment. The COMBINED treatment also demonstrated significantly increased ipsilateral left parietal theta when emotionally threatening images were presented in the left visual field. This is suggestive of increased attentional processing of non-emotionally threatening stimuli which may indicate a general increase in attentional processing function in the COMBINED treatment irrespective of the affective content of stimuli.

Evidence of increased attentional processing capacity in the COMBINED treatment during the attention bias task was not supported by the behavioural data which demonstrated no significant or meaningful (21) difference between treatments in responsiveness to stimuli. It is important to note the observed increased theta activity relates to the early processing of the attentional bias task images. The heightened theta peaked immediately upon viewing the images (0.5–0.75 ms) and subsided as the trial progressed. Therefore, this activity related to early attentional processing of emotionally threatening images may not have been sufficient to impact upon behavioural responses which were temporally removed from initial attentional processing-related theta activation. The stimulus latency employed on the attentional bias task (500 ms) was specifically selected as shorter stimulus exposure times (≤150 ms) have been shown to be less sensitive to demonstration of a relationship between threat processing and theta activity (40). However, a reduction in the delay between stimulus presentation and behavioural response (≥150–≤500 ms) may increase the likelihood of demonstrating any behavioural effects of the heightened attentional capacity suggested by the theta response in the COMBINED treatment.

Spectral EEG analysis during the attentional bias task also demonstrated significantly higher alpha band activity in left and right parietal regions (and ipsilateral in the left parietal cortex) in the COMBINED treatment compared to the PLACEBO treatment. This significance did not follow through into the *a priori* ROI analysis; however, it is evident from Figure 6 that the whole brain results are driven by a larger contra and ipsilateral decrease in alpha band activity in the PLACEBO group, compared to all other treatments and particularly the COMBINED group treatment. Decreased alpha power has traditionally been associated with increased task engagement, for example heightened perceptual judgement or attentiveness (41, 42). The degree of suppression of alpha is considered to index the level of task demand or relevance (30, 43–45). Whilst the significance of alpha modulation by the COMBINED treatment did not retain significance in the ROI analysis, the mean alpha power values indicate that this difference was predominantly the result of a decrease in alpha power in the PLACEBO treatment being attenuated in the COMBINED treatment, and to a lesser extent RHODIOLA and GREEN TEA treatments. This is suggestive of a greater level of attentional engagement with the stress imagery in the PLACEBO treatment. However, the proposed relationship between alpha inhibition and attentional engagement is largely reflective of the operation of non-affective attentional processes; the relationship between alpha activity and affective attentional processes is inconsistent and at times contradictory (46), including evidence of no modulation of alpha power (47, 48), increased alpha power (49, 50) and decreased alpha power (51, 52) during the processing of affective stimuli. Therefore, the lack of significant ROI treatment differences and the inconsistent pattern of relationship between affective attentional processing and alpha activity limits further interpretation of the findings.

### Attention Switching Event-Related Potential

No significant effects of treatment on attention related ERPs during the attention switching task were found. The lack of significant treatment differences for specific attention-related ERPs during this task is perhaps not surprising considering the lack of behavioural performance differentiation between treatments. The enhanced spectral EEG activity observed in the COMBINED treatment may therefore represent a general increase in attentional functional capacity rather than relating to specific indices of attentional processing on the attention switching task that would be expected to be shown in the ERP data, and further reflected in behavioural performance outputs. The lack of any treatment effects may also be due to the absence of the well-characterised differentiation between switch, nested and pre-switch trials typically observed in this attention switching paradigm. This lack of differentiation between trial types may be as a result of this attention switching task being performed under conditions of stress; a context in which, to the authors’ knowledge, the characteristic ERP response for this task has yet to be confirmed.

### Limitations and Future Directions

We acknowledge the proposed enhanced attentional capacity suggested by the modulation of theta activity during the employed attentional tasks was not supported by the behavioural or ERP data. This disassociation between EGG spectral activity proposed to index enhanced neural functional capacity and observed behavioural performance is not uncommon in the literature [e.g., (1, 9)]. However, behavioural confirmation of the observed EEG effects of the COMBINED treatment would strengthen the evidence of the potential cognitive enhancing effects of this combination of ingredients. The employed tests may not have been sensitive enough to capture any behavioural improvements associated with the enhanced cognitive capacity indicated by the EEG data. However, both employed tasks have been widely proven to be sensitive to the effects of stress and extensively applied and well characterised in EEG experimentation. Future attempts to confirm the potential for this combination of ingredients to enhance attentional performance should consider the use of a multi-modal visuo-spatial attentional switching task, and vary the delay between exposure to emotionally threatening images and behavioural response for the attentional bias task.

Timing of exposure to the attentional tasks may have been a potential factor in the lack of observed effects on behavioural performance. Impaired cognitive performance under stress can often occur only during the period when cortisol and sympathetic arousal are concurrently activated (53, 54). We have previously reported that the cortisol response was still elevated during completion of the attentional tasks, but the sympathetic response had returned to baseline levels (5). The timing of cognitive testing was constrained by the methodological requirements of the EEG, however, cognitive effects may be more likely demonstrated if cognitive testing is undertaken during peak sympathetic and cortisol activation. Increasing the sensitivity of the attentional tasks to the modulating effects of acute stress may increase the likelihood that the enhanced theta activation observed has more room to confer protective effects on detriments in performance.

Future studies of the observed effects should consider recording dietary intake prior to participation to assess any impact of basal dietary status on measured outcomes; for example, via the completion of a daily food intake diary. However, the accuracy of self-reported dietary intake recall is often questioned, especially when considering the small margins of scale in levels of available vitamin, minerals and botanicals relevant here. Biochemical assessment of baseline levels would be required to give a more meaningful insight into baseline levels. However, this was not feasible in a trial of this size.

### Conclusion

We further demonstrate the combination of green tea and rhodiola extract, magnesium and a B vitamin complex (B_6_, B_9_, and B_12_) offers potential functional benefits under conditions of stress. This combination of ingredients has previously been demonstrated to induce a relaxed, attentive state (indexed by increased resting theta activity) and attenuate subjective stress and anxiety under conditions of acute stress (5). Here we further demonstrate the capacity of these ingredients to modulate, predominantly, theta activity during the execution of two distinct attentional tasks. These findings suggest increased attentional capacity following the intake of the COMBINED treatment. Further examination of these ingredients in relation to attentional performance is warranted to demonstrate the potential of this heightened attentional capacity to protect or enhance behavioural performance under condition of stress.

## Data Availability Statement

The datasets presented in this article are not readily available. Due to its proprietary nature the study data cannot be made openly available. Requests to access the datasets should be directed to the corresponding author.

## Ethics Statement

The studies involving human participants were reviewed and approved by the School of Psychology Research Ethics Committee, University of Leeds. The patients/participants provided their written informed consent to participate in this study.

## Author Contributions

All authors listed have made a substantial, direct, and intellectual contribution to the work, and approved it for publication.

## Conflict of Interest

LD has received consultancy and honoraria for work in the area of stress from Sanofi. The remaining authors declare that the research was conducted in the absence of any commercial or financial relationships that could be construed as a potential conflict of interest.

## Publisher’s Note

All claims expressed in this article are solely those of the authors and do not necessarily represent those of their affiliated organizations, or those of the publisher, the editors and the reviewers. Any product that may be evaluated in this article, or claim that may be made by its manufacturer, is not guaranteed or endorsed by the publisher.

## Footnotes

1
www.random.org
